# First record of *Arionvulgaris* Moquin-Tandon, 1855 (Arionidae) from Armenia

**DOI:** 10.3897/BDJ.12.e121176

**Published:** 2024-04-08

**Authors:** Meri Arzumanyan, Gohar Zhamakochyan, Hasmik Torosyan, Arevik Ghrmajyan, Marine Arakelyan, Siranush Nanagulyan, Lusine Margaryan, Sargis Aghayan, Robert B Davis, Ágnes Turóci

**Affiliations:** 1 Department of Zoology, Yerevan State University, Alex Manoogian 1, 0025, Yerevan, Armenia Department of Zoology, Yerevan State University, Alex Manoogian 1, 0025 Yerevan Armenia; 2 Department of Botany and Mycology, Yerevan State University, Alex Manoogian 1, 0025, Yerevan, Armenia Department of Botany and Mycology, Yerevan State University, Alex Manoogian 1, 0025 Yerevan Armenia; 3 Laboratory of Molecular Parasitology, Scientific Center of Zoology and Hydroecology, NAS RA., P. Sevak 7, 0014, Yerevan, Armenia Laboratory of Molecular Parasitology, Scientific Center of Zoology and Hydroecology, NAS RA., P. Sevak 7, 0014 Yerevan Armenia; 4 Department of Zoology, Institute of Ecology and Earth Sciences, University of Tartu, J. Liivi 2, 50409, Tartu, Estonia Department of Zoology, Institute of Ecology and Earth Sciences, University of Tartu, J. Liivi 2, 50409 Tartu Estonia; 5 Plant Protection Institute, Centre for Agricultural Research, HUN–REN, Herman Ottó út 15, H–1022, Budapest, Hungary Plant Protection Institute, Centre for Agricultural Research, HUN–REN, Herman Ottó út 15, H–1022 Budapest Hungary

**Keywords:** Gastropoda, biological invasions, Caucasus, terrestrial molluscs

## Abstract

**Background:**

*Arionvulgaris* Moquin-Tandon, 1855 is amongst the fastest-spreading terrestrial slugs Europe-wide. In recent years, it has been recorded in Canada, Mexico and continues to expand eastwards into Eurasia. Renowned for its high invasiveness, combatting its swift spread creates significant challenges in organising effective preventative measures.

**New information:**

This study presents the first record of *Arionvulgaris* from Armenia, which is the second record of this species' invasion of the Caucasus. In 2022, a substantial population of *A.vulgaris* was observed close to the City of Stepanavan, which is also the first record in Armenia of the family Arionidae. How the species was introduced to Armenia remains unknown. Identification of *Arionvulgaris* was conducted, based on external and genital morphology and mitochondrial CO1 (cytochrome c oxidase subunit 1) gene sequencing, revealing notable similarities with Central European clades. Our results confirm the introduction and distribution of *A.vulgaris* to Armenia. Invasion of such species into Armenia will require additional monitoring and would be aided by further research on Armenia’s mollusc fauna in the future.

## Introduction

*Arionvulgaris* Moquin–Tandon, 1855 stands prominently as one of Europe's most notorious pests, included in a list of the 100 most dangerous invasive species of Europe (Rabitsch 2009). The native range of *A.vulgaris* is considered somewhere from southwest France to southwest Germany (Pfenninger et al. 2014, Zemanova et al. 2016, Zając et al. 2019).

*Arionvulgaris* feeds on vegetation in gardens and orchards and causes serious agricultural damage (Grimm et al. 2000). This pest is characterised by a large body size, high reproductive capacity and survival in adverse conditions (Kozłowski 2007). In addition, *A.vulgaris* might not have natural enemies in newly-invaded territories, but a parasitic nematode species (*Phasmarhabditishermaphrodita*) can infect slugs, which might be useful for biological control (Iglesias et al. 2001, Rae et al. 2007, Kozłowski et al. 2014). Some predatory beetles may also be effective against them (Hatteland 2010, Hatteland et al. 2010, Hatteland et al. 2011). Individuals live for approximately one year, but some may live for two years. Individuals become mature in spring or summer. The mating period usually starts in June and may last until December, depending on climatic conditions (Kozłowski 2007). Egg-laying reaches its peak around August or September. Most adults die in autumn after reproduction. Young individuals hatch in late autumn and then enter the soil and overwinter (Davies 1987, Kozłowski 2007, Hatteland et al. 2013).

The species is currently invasive in several countries and has also been found in the Caucasus Region (Schikov and Komarov 2021). The first record of this species in the Caucasus was in north Ossetia–Alania in 2019 (Schikov and Komarov 2021). However, the first record from Russia was in 2009 in gardens in Moscow (Schikov 2016). Additionally, it was reported from Türkiye in 2017 in the City of Isparta (Yildırım and Gürlek 2017), but this observation needs to be verified because only the external appearance was examined (Reise et al. 2018).

This study documents the species' occurrence in Armenia for the first time.

## Materials and methods

### Sampling

Species sampling was performed from the beginning of September in 2022, in Gyulagarak Village, Lori Province, northern Armenia. Sampling was repeated during the summer season of 2023 and over 30 specimens of *Arionvulgaris* were sampled from the same area. Individuals were in high abundance in vegetable gardens, where potatoes, tomatoes, cucumbers and other vegetables were usually planted by the local people. The species distribution is considered to be the areas surrounding Gyulagarak Village and Stepanavan City. An individual of a species resembling *A.vulgaris* was found in a forested area in the City of Dilijan by Prof. S. Pipoyan. However, due to the mixed forest habitat and the fact that there was neither genetic nor thorough anatomical proof that this was an *A.vulgaris* individual, we do not include Dilijan as a distribution point for the species at this time.

Sampling in Gyulagarak was performed by hand and individuals were kept alive until they were transported to the Yerevan State University, Laboratory of Invertebrate Zoology. Specimens for sequencing were immersed in 70% ethanol and later transferred to 96% ethanol for better preservation. The ethanol was changed until the point at which no more discoloration occurred. Specimens for dissection were preserved by submerging them in a 70% ethanol solution, ensuring the fixation of the specimens for subsequent analysis.

A total of thirteen specimens were used for this study: nine vouchers were used for sequencing and three were used for dissection. Specimen CaBOL-1013790 was used to illustrate the external morphology of the species. The three dissected ethanol-preserved voucher specimens are deposited in the Hungarian Natural History Museum, Budapest (inventory numbers: HNHM 105451a, HNHM 105451b and HNHM 105451c). Specimens CaBOL-1013789, CaBOL-1013790, CaBOL-1013791, CaBOL-1013792, CaBOL-1013793, CaBOL-1013794, CaBOL-1013795, CaBOL-1013796 and CaBOL-1013797 were used for the sequencing. The specimens that were used for sequencing are part of the Malacological collection of the Department of Zoology at Yerevan State University, stored under the institute museum ID: YSU_MOL_MA.

### Morphology, dissection and photography

Examination of external and genital morphological traits was based on the works of Wiktor (1983), Rowson et al. (2014) and Reise et al. (2020).

Dissection was performed under a Zeiss Stemi 305 stereomicroscope. The genital organs of the slugs were photographed using a Canon EOS 2000d camera with Tamron SP AF 90 mm F/2.8 Di MACRO 1:1 macro objective lens. One camera-mounted flash with two studio flash units (BlitzBirne Mikrosat) were used on the left and right side of the subject, respectively, using white umbrellas for reflection.

### DNA extraction, PCR and sequencing

The total genomic DNA was extracted from each sample using a HiGene™ Genomic DNA Prep Kit (For Whole Blood, Bacterium, Plant, Animal Tissue, Fungus), following the manufacturer’s protocol (catalogue number: GD141-100, BIOFACT Co., Ltd., Daejeon, Korea).

Partial sequences of the cytochrome oxidase subunit 1 (CO1) were amplified via polymerase chain reaction (PCR), employing the primer pairs LCOI490-JJ 5'-GGTCAACAAATCATAAAGATATTGG and HCO2198-JJ 5'-TAAACTTCAGGGTGACCAAAAAATCA (Folmer et al. 1994). The thermal conditions comprised initial denaturation at 95°C for 1 minute, followed by the first cycle set (15 cycles) of 94°C for 30 seconds, annealing at 55°C for 1 minute (decreasing by 1°C per cycle) and extension at 72°C for 1 minute and 30 seconds. The second cycle set (25 cycles) involved steps of 94°C for 35 seconds, 45°C for 1 minute, and 72°C for 1 minute and 30 seconds, followed by a single cycle at 72°C for 3 minutes and a final extension step at 72°C for 5 minutes, followed by indefinite hold at 4°C. PCR amplicons were observed on 1.5% agarose gels with 2 μl of PCR product. Sequencing of the unpurified PCR products in both directions was carried out at the Beijing Genomics Institute (Hong Kong, CN) using the amplification primers.

### Sequence processing

Analysis of nine sequences of specimens from garden sites, (sequence length 655 bp), as well as the quality check of the sequences, was conducted using Geneious Prime 2023.2.1 (http://www.geneious.com). The haplotype data file was generated using DnaSP 6 (Rozas et al. 2017) and resulted in three unique haplotypes. A sequence similarity search was conducted using the BLAST tool of NCBI directly in Geneous Prime 2023.2.1. The most closely related lineages of *A.vulgaris* were extracted and aligned with our haplotypes. A Neighbour-joining tree (genetic distance model: Tamura–Nei) was constructed to observe the relationship of both our and previously found haplotypes (Nei and Kumar 2000, Tamura et al. 2004).

The GenBank IDs of the nine voucher specimens used in this research are as follows: CaBOL-1013797:PP465469, CaBOL-1013796:PP465470, CaBOL-1013795:PP465471, CaBOL-1013794:PP465472, CaBOL-1013793:PP465473, CaBOL-1013792:PP465474, CaBOL-1013791:PP465475, CaBOL-1013790:PP465476, CaBOL-1013789:PP465477.

## Taxon treatments

### Arion vulgaris

Moquin-Tandon, 1855

A409340B-03A3-5319-BF4D-32B1F7F96B87

#### Materials

- **Type status:** Other material. **Occurrence:** catalogNumber: HNHM 105451a; recordedBy: Meri Arzumanyan; individualCount: 1; sex: hermaphrodite; lifeStage: subadult; occurrenceID: 8DC67D2A-7463-5B91-AD92-866FA1C4F7EE; **Taxon:** scientificName: Arionvulgaris; acceptedNameUsage: Spanish slug; originalNameUsage: Arionvulgaris; nameAccordingTo: sensu; higherClassification: Eukaryota; kingdom: Animalia; phylum: Mollusca; class: Gastropoda; order: Stylommatophora; family: Arionidae; genus: Arion; specificEpithet: vulgaris; taxonRank: species; scientificNameAuthorship: Moquin-Tandon, 1855; vernacularName: Spanish slug; taxonomicStatus: accepted; **Location:** locationID: Stepanavan; higherGeography: Lesser Caucasus; continent: Eurasia; country: Armenia; countryCode: ARM; stateProvince: Lori; locality: Gyulagarak village, Lori province, ARM; decimalLatitude: 40.9686; decimalLongitude: 44.4675; georeferenceProtocol: GPS; **Identification:** identifiedBy: Meri Arzumanyan; dateIdentified: 08-08-23; identificationQualifier: Dissection; **Event:** samplingProtocol: hand sampling; habitat: garden; fieldNumber: Arion_001; fieldNotes: First record of invasion; **Record Level:** language: Armenian; collectionID: HNHM 105451a; datasetID: HNHM; institutionCode: YSU_MOL_MA; collectionCode: Molluscs; basisOfRecord: PreservedSpecimen
- **Type status:** Other material. **Occurrence:** catalogNumber: HNHM 105451b; recordedBy: Meri Arzumanyan; individualCount: 1; sex: hermaphrodite; lifeStage: subadult; occurrenceID: 9DF99457-A965-56DC-9DC9-11FC16B6E43F; **Taxon:** scientificName: Arionvulgaris; acceptedNameUsage: Spanish slug; originalNameUsage: Arionvulgaris; nameAccordingTo: sensu; higherClassification: Eukaryota; kingdom: Animalia; phylum: Mollusca; class: Gastropoda; order: Stylommatophora; family: Arionidae; genus: Arion; specificEpithet: vulgaris; taxonRank: species; scientificNameAuthorship: Moquin-Tandon, 1855; vernacularName: Spanish slug; taxonomicStatus: accepted; **Location:** locationID: Stepanavan; higherGeography: Lesser Caucasus; continent: Eurasia; country: Armenia; countryCode: ARM; stateProvince: Lori; locality: Gyulagarak village, Lori province, ARM; decimalLatitude: 40.9686; decimalLongitude: 44.4675; georeferenceProtocol: GPS; **Identification:** identifiedBy: Meri Arzumanyan; dateIdentified: 08-08-23; identificationQualifier: Dissection; **Event:** samplingProtocol: hand sampling; habitat: garden; fieldNumber: Arion_001; fieldNotes: First record of invasion; **Record Level:** language: Armenian; collectionID: HNHM 105451b; datasetID: HNHM; institutionCode: YSU_MOL_MA; collectionCode: Molluscs; basisOfRecord: PreservedSpecimen
- **Type status:** Other material. **Occurrence:** catalogNumber: HNHM 105451c; recordedBy: Meri Arzumanyan; individualCount: 1; sex: hermaphrodite; lifeStage: juvenile; occurrenceID: AEE31151-C361-5C48-A8BD-7062A2E3F3A8; **Taxon:** scientificName: Arionvulgaris; acceptedNameUsage: Spanish slug; originalNameUsage: Arionvulgaris; nameAccordingTo: sensu; higherClassification: Eukaryota; kingdom: Animalia; phylum: Mollusca; class: Gastropoda; order: Stylommatophora; family: Arionidae; genus: Arion; specificEpithet: vulgaris; taxonRank: species; scientificNameAuthorship: Moquin-Tandon, 1855; vernacularName: Spanish slug; taxonomicStatus: accepted; **Location:** locationID: Stepanavan; higherGeography: Lesser Caucasus; continent: Eurasia; country: Armenia; countryCode: ARM; stateProvince: Lori; locality: Gyulagarak village, Lori province, ARM; decimalLatitude: 40.9686; decimalLongitude: 44.4675; georeferenceProtocol: GPS; **Identification:** identifiedBy: Meri Arzumanyan; dateIdentified: 08-08-23; identificationQualifier: Dissection; **Event:** samplingProtocol: hand sampling; habitat: garden; fieldNumber: Arion_001; fieldNotes: First record of invasion; **Record Level:** language: Armenian; collectionID: HNHM 105451c; datasetID: HNHM; institutionCode: YSU_MOL_MA; collectionCode: Molluscs; basisOfRecord: PreservedSpecimen
- **Type status:** Other material. **Occurrence:** catalogNumber: CaBOL-1013789; recordedBy: Meri Arzumanyan; individualCount: 1; sex: hermaphrodite; occurrenceID: 2F28EE20-0497-58C4-A62D-7BB43361BEA9; **Taxon:** scientificName: Arionvulgaris; acceptedNameUsage: Spanish slug; originalNameUsage: Arionvulgaris; nameAccordingTo: sensu; higherClassification: Eukaryota; kingdom: Animalia; phylum: Mollusca; class: Gastropoda; order: Stylommatophora; family: Arionidae; genus: Arion; specificEpithet: vulgaris; taxonRank: species; scientificNameAuthorship: Moquin-Tandon, 1855; vernacularName: Spanish slug; taxonomicStatus: accepted; **Location:** locationID: Stepanavan; higherGeography: Lesser Caucasus; continent: Eurasia; country: Armenia; countryCode: ARM; stateProvince: Lori; locality: Gyulagarak village, Lori province, ARM; decimalLatitude: 40.9686; decimalLongitude: 44.4675; georeferenceProtocol: GPS; **Identification:** identifiedBy: Meri Arzumanyan; dateIdentified: 09-12-22; identificationQualifier: Sequence; **Event:** samplingProtocol: hand sampling; habitat: garden; fieldNumber: Arion_001; fieldNotes: First record of invasion; **Record Level:** language: Armenian; collectionID: CaBOL-1013789; datasetID: CaBOL; institutionCode: YSU_MOL_MA; collectionCode: Molluscs; basisOfRecord: PreservedSpecimen
- **Type status:** Other material. **Occurrence:** catalogNumber: CaBOL-1013790; recordedBy: Meri Arzumanyan; individualCount: 1; sex: hermaphrodite; occurrenceID: 8C474C5B-2524-5391-83FE-BDFC3FFC931A; **Taxon:** scientificName: Arionvulgaris; acceptedNameUsage: Spanish slug; originalNameUsage: Arionvulgaris; nameAccordingTo: sensu; higherClassification: Eukaryota; kingdom: Animalia; phylum: Mollusca; class: Gastropoda; order: Stylommatophora; family: Arionidae; genus: Arion; specificEpithet: vulgaris; taxonRank: species; scientificNameAuthorship: Moquin-Tandon, 1855; vernacularName: Spanish slug; taxonomicStatus: accepted; **Location:** locationID: Stepanavan; higherGeography: Lesser Caucasus; continent: Eurasia; country: Armenia; countryCode: ARM; stateProvince: Lori; locality: Gyulagarak village, Lori province, ARM; decimalLatitude: 40.9686; decimalLongitude: 44.4675; georeferenceProtocol: GPS; **Identification:** identifiedBy: Meri Arzumanyan; dateIdentified: 07-01-23; identificationQualifier: Sequence; **Event:** samplingProtocol: hand sampling; habitat: garden; fieldNumber: Arion_001; fieldNotes: First record of invasion; **Record Level:** language: Armenian; collectionID: CaBOL-1013790; datasetID: CaBOL; institutionCode: YSU_MOL_MA; collectionCode: Molluscs; basisOfRecord: PreservedSpecimen
- **Type status:** Other material. **Occurrence:** catalogNumber: CaBOL-1013791; recordedBy: Meri Arzumanyan; individualCount: 1; sex: hermaphrodite; occurrenceID: 41A510F9-AE0E-58AA-BBCB-7EF286C982A0; **Taxon:** scientificName: Arionvulgaris; acceptedNameUsage: Spanish slug; originalNameUsage: Arionvulgaris; nameAccordingTo: sensu; higherClassification: Eukaryota; kingdom: Animalia; phylum: Mollusca; class: Gastropoda; order: Stylommatophora; family: Arionidae; genus: Arion; specificEpithet: vulgaris; taxonRank: species; scientificNameAuthorship: Moquin-Tandon, 1855; vernacularName: Spanish slug; taxonomicStatus: accepted; **Location:** locationID: Stepanavan; higherGeography: Lesser Caucasus; continent: Eurasia; country: Armenia; countryCode: ARM; stateProvince: Lori; locality: Gyulagarak village, Lori province, ARM; decimalLatitude: 40.9686; decimalLongitude: 44.4675; georeferenceProtocol: GPS; **Identification:** identifiedBy: Meri Arzumanyan; dateIdentified: 07-01-23; identificationQualifier: Sequence; **Event:** samplingProtocol: hand sampling; habitat: garden; fieldNumber: Arion_001; fieldNotes: First record of invasion; **Record Level:** language: Armenian; collectionID: CaBOL-1013791; datasetID: CaBOL; institutionCode: YSU_MOL_MA; collectionCode: Molluscs; basisOfRecord: PreservedSpecimen
- **Type status:** Other material. **Occurrence:** catalogNumber: CaBOL-1013792; recordedBy: Meri Arzumanyan; individualCount: 1; sex: hermaphrodite; occurrenceID: AA9DA143-4568-5355-AA23-A89C3E62CF8C; **Taxon:** scientificName: Arionvulgaris; acceptedNameUsage: Spanish slug; originalNameUsage: Arionvulgaris; nameAccordingTo: sensu; higherClassification: Eukaryota; kingdom: Animalia; phylum: Mollusca; class: Gastropoda; order: Stylommatophora; family: Arionidae; genus: Arion; specificEpithet: vulgaris; taxonRank: species; scientificNameAuthorship: Moquin-Tandon, 1855; vernacularName: Spanish slug; taxonomicStatus: accepted; **Location:** locationID: Stepanavan; higherGeography: Lesser Caucasus; continent: Eurasia; country: Armenia; countryCode: ARM; stateProvince: Lori; locality: Gyulagarak village, Lori province, ARM; decimalLatitude: 40.9686; decimalLongitude: 44.4675; georeferenceProtocol: GPS; **Identification:** identifiedBy: Meri Arzumanyan; dateIdentified: 07-01-23; identificationQualifier: Sequence; **Event:** samplingProtocol: hand sampling; habitat: garden; fieldNumber: Arion_001; fieldNotes: First record of invasion; **Record Level:** language: Armenian; collectionID: CaBOL-1013792; datasetID: CaBOL; institutionCode: YSU_MOL_MA; collectionCode: Molluscs; basisOfRecord: PreservedSpecimen
- **Type status:** Other material. **Occurrence:** catalogNumber: CaBOL-1013793; recordedBy: Meri Arzumanyan; individualCount: 1; sex: hermaphrodite; occurrenceID: 3F851C25-634C-518D-906B-BD0EF6E01698; **Taxon:** scientificName: Arionvulgaris; acceptedNameUsage: Spanish slug; originalNameUsage: Arionvulgaris; nameAccordingTo: sensu; higherClassification: Eukaryota; kingdom: Animalia; phylum: Mollusca; class: Gastropoda; order: Stylommatophora; family: Arionidae; genus: Arion; specificEpithet: vulgaris; taxonRank: species; scientificNameAuthorship: Moquin-Tandon, 1855; vernacularName: Spanish slug; taxonomicStatus: accepted; **Location:** locationID: Stepanavan; higherGeography: Lesser Caucasus; continent: Eurasia; country: Armenia; countryCode: ARM; stateProvince: Lori; locality: Gyulagarak village, Lori province, ARM; decimalLatitude: 40.9686; decimalLongitude: 44.4675; georeferenceProtocol: GPS; **Identification:** identifiedBy: Meri Arzumanyan; dateIdentified: 08-08-23; identificationQualifier: Sequence; **Event:** samplingProtocol: hand sampling; habitat: garden; fieldNumber: Arion_001; fieldNotes: First record of invasion; **Record Level:** language: Armenian; collectionID: CaBOL-1013793; datasetID: CaBOL; institutionCode: YSU_MOL_MA; collectionCode: Molluscs; basisOfRecord: PreservedSpecimen
- **Type status:** Other material. **Occurrence:** catalogNumber: CaBOL-1013794; recordedBy: Meri Arzumanyan; individualCount: 1; sex: hermaphrodite; occurrenceID: 20BF850A-EBAE-5974-AF91-CACD7F6D0F1A; **Taxon:** scientificName: Arionvulgaris; acceptedNameUsage: Spanish slug; originalNameUsage: Arionvulgaris; nameAccordingTo: sensu; higherClassification: Eukaryota; kingdom: Animalia; phylum: Mollusca; class: Gastropoda; order: Stylommatophora; family: Arionidae; genus: Arion; specificEpithet: vulgaris; taxonRank: species; scientificNameAuthorship: Moquin-Tandon, 1855; vernacularName: Spanish slug; taxonomicStatus: accepted; **Location:** locationID: Stepanavan; higherGeography: Lesser Caucasus; continent: Eurasia; country: Armenia; countryCode: ARM; stateProvince: Lori; locality: Gyulagarak village, Lori province, ARM; decimalLatitude: 40.9686; decimalLongitude: 44.4675; georeferenceProtocol: GPS; **Identification:** identifiedBy: Meri Arzumanyan; dateIdentified: 08-08-23; identificationQualifier: Sequence; **Event:** samplingProtocol: hand sampling; habitat: garden; fieldNumber: Arion_001; fieldNotes: First record of invasion; **Record Level:** language: Armenian; collectionID: CaBOL-1013794; datasetID: CaBOL; institutionCode: YSU_MOL_MA; collectionCode: Molluscs; basisOfRecord: PreservedSpecimen
- **Type status:** Other material. **Occurrence:** catalogNumber: CaBOL-1013795; recordedBy: Meri Arzumanyan; individualCount: 1; sex: hermaphrodite; occurrenceID: A99D5774-E829-59EE-9051-C66146807805; **Taxon:** scientificName: Arionvulgaris; acceptedNameUsage: Spanish slug; originalNameUsage: Arionvulgaris; nameAccordingTo: sensu; higherClassification: Eukaryota; kingdom: Animalia; phylum: Mollusca; class: Gastropoda; order: Stylommatophora; family: Arionidae; genus: Arion; specificEpithet: vulgaris; taxonRank: species; scientificNameAuthorship: Moquin-Tandon, 1855; vernacularName: Spanish slug; taxonomicStatus: accepted; **Location:** locationID: Stepanavan; higherGeography: Lesser Caucasus; continent: Eurasia; country: Armenia; countryCode: ARM; stateProvince: Lori; locality: Gyulagarak village, Lori province, ARM; decimalLatitude: 40.9686; decimalLongitude: 44.4675; georeferenceProtocol: GPS; **Identification:** identifiedBy: Meri Arzumanyan; dateIdentified: 08-08-23; identificationQualifier: Sequence; **Event:** samplingProtocol: hand sampling; habitat: garden; fieldNumber: Arion_001; fieldNotes: First record of invasion; **Record Level:** language: Armenian; collectionID: CaBOL-1013795; datasetID: CaBOL; institutionCode: YSU_MOL_MA; collectionCode: Molluscs; basisOfRecord: PreservedSpecimen
- **Type status:** Other material. **Occurrence:** catalogNumber: CaBOL-1013796; recordedBy: Meri Arzumanyan; individualCount: 1; sex: hermaphrodite; occurrenceID: 99DC1334-2758-5445-93BB-5B44804D06C0; **Taxon:** scientificName: Arionvulgaris; acceptedNameUsage: Spanish slug; originalNameUsage: Arionvulgaris; nameAccordingTo: sensu; higherClassification: Eukaryota; kingdom: Animalia; phylum: Mollusca; class: Gastropoda; order: Stylommatophora; family: Arionidae; genus: Arion; specificEpithet: vulgaris; taxonRank: species; scientificNameAuthorship: Moquin-Tandon, 1855; vernacularName: Spanish slug; taxonomicStatus: accepted; **Location:** locationID: Stepanavan; higherGeography: Lesser Caucasus; continent: Eurasia; country: Armenia; countryCode: ARM; stateProvince: Lori; locality: Gyulagarak village, Lori province, ARM; decimalLatitude: 40.9686; decimalLongitude: 44.4675; georeferenceProtocol: GPS; **Identification:** identifiedBy: Meri Arzumanyan; dateIdentified: 08-08-23; identificationQualifier: Sequence; **Event:** samplingProtocol: hand sampling; habitat: garden; fieldNumber: Arion_001; fieldNotes: First record of invasion; **Record Level:** language: Armenian; collectionID: CaBOL-1013796; datasetID: CaBOL; institutionCode: YSU_MOL_MA; collectionCode: Molluscs; basisOfRecord: PreservedSpecimen
- **Type status:** Other material. **Occurrence:** catalogNumber: CaBOL-1013797; recordedBy: Meri Arzumanyan; individualCount: 1; sex: hermaphrodite; occurrenceID: 0584E305-B066-58AE-90AF-05A73BE2DB4B; **Taxon:** scientificName: Arionvulgaris; acceptedNameUsage: Spanish slug; originalNameUsage: Arionvulgaris; nameAccordingTo: sensu; higherClassification: Eukaryota; kingdom: Animalia; phylum: Mollusca; class: Gastropoda; order: Stylommatophora; family: Arionidae; genus: Arion; specificEpithet: vulgaris; taxonRank: species; scientificNameAuthorship: Moquin-Tandon, 1855; vernacularName: Spanish slug; taxonomicStatus: accepted; **Location:** locationID: Stepanavan; higherGeography: Lesser Caucasus; continent: Eurasia; country: Armenia; countryCode: ARM; stateProvince: Lori; locality: Gyulagarak village, Lori province, ARM; decimalLatitude: 40.9686; decimalLongitude: 44.4675; georeferenceProtocol: GPS; **Identification:** identifiedBy: Meri Arzumanyan; dateIdentified: 08-08-23; identificationQualifier: Sequence; **Event:** samplingProtocol: hand sampling; habitat: garden; fieldNumber: Arion_001; fieldNotes: First record of invasion; **Record Level:** language: Armenian; collectionID: CaBOL-1013797; datasetID: CaBOL; institutionCode: YSU_MOL_MA; collectionCode: Molluscs; basisOfRecord: PreservedSpecimen

#### Description

**Figure 1.**
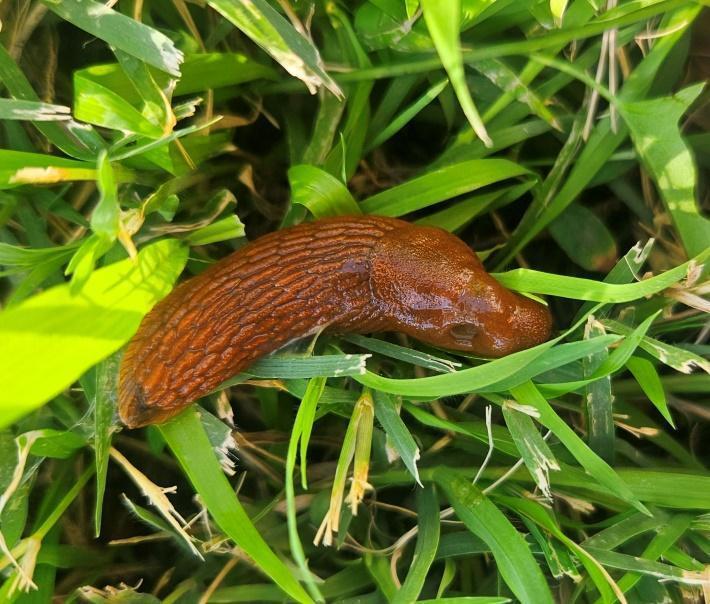
*Arionvulgaris* specimen from Gyulagarak (CaBOL-1013790). The photograph was taken by M. Arzumanyan.

Adult living specimens were 70–110 mm long when fully extended. Collected individuals were unicolour brown with an orange hue without bands, the tubercles were coarse, the foot fringe was orange or brown (same shade as body colour) with thin vertical black lines, the head had an orange tint and the tentacles were black (Fig. 1).

**Figure 2.**
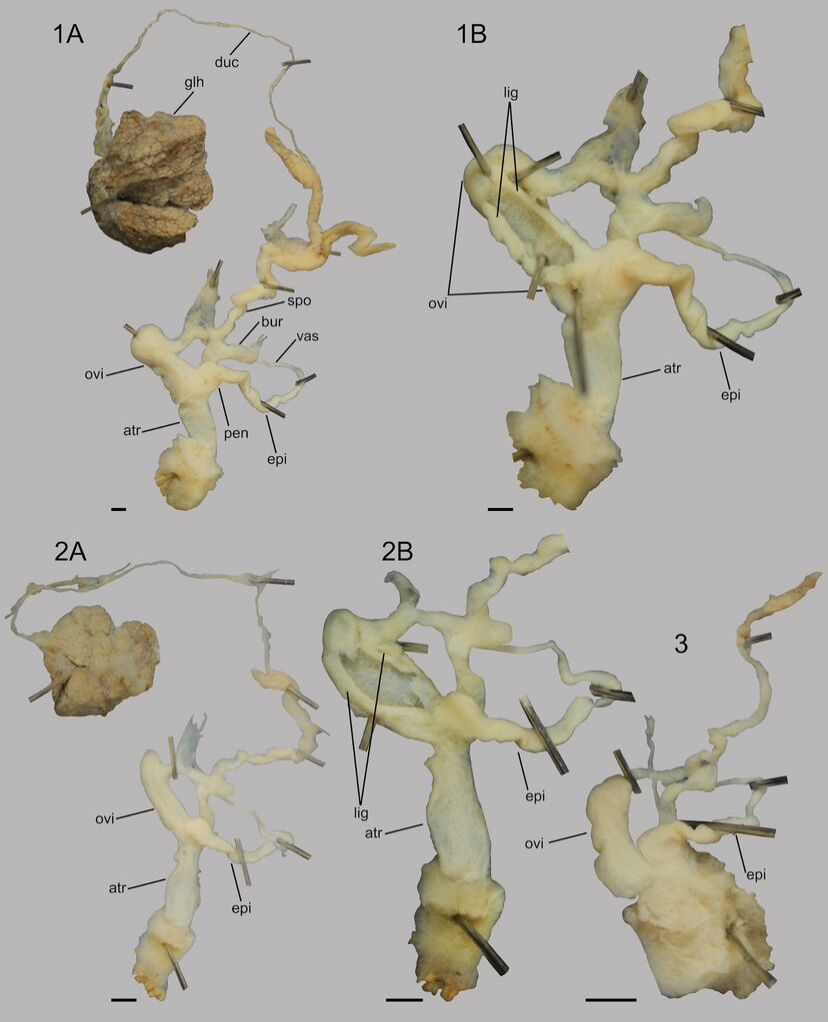
Genital tract of three specimens deposited in the Natural History Museum of Budapest: 1: HNHM105451a, 2: HNHM105451b, 3: HNHM105451c. 1B and 2B: same specimens as in A with oviduct opened 1A: Abbreviations: atr = atrium, bur = bursa copulatrix, duc = ductus hermaphroditicus, epi = epiphallus, glh = glandula hermaphroditica, pen = penis, lig = ligula, ovi = oviductus, spo = spermoviductus. All scale bars = 1 mm.

Samples for dissection were collected during the summer of 2023. Although all three dissected specimens were subadults or juveniles, the main genital characters were visible. The main distinctive character is the dilated anterior (i.e. closer to the genital pore) part of the oviduct, which contains a longitudinal ligula that has two elongated flanks. The posterior part of the oviduct is short and not dilated. The bursa is somewhat elongated and almost as wide as their ducts. The vas deferens is short and the epiphallus is slightly wider than the vas deferens in all cases (Fig. 2).

#### Distribution

**Figure 3.**
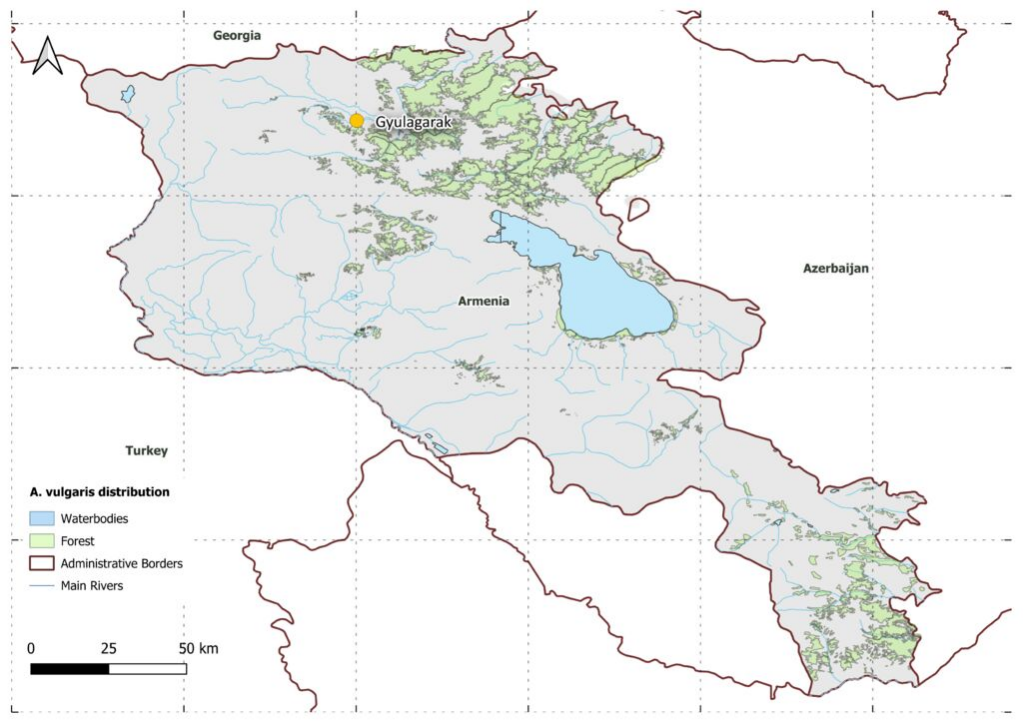
Sampling locations of *Arionvulgaris* in Armenia.

The map of Armenia presented in Fig. 3 highlights the specific sampling locations where *Arionvulgaris* was recorded. In 2022, the species was documented for the first time in Armenia in Gyulagarak.

#### Taxon discussion

First record of *Arionvulgaris* in Armenia.

#### Molecular analyses

**Figure 4.**
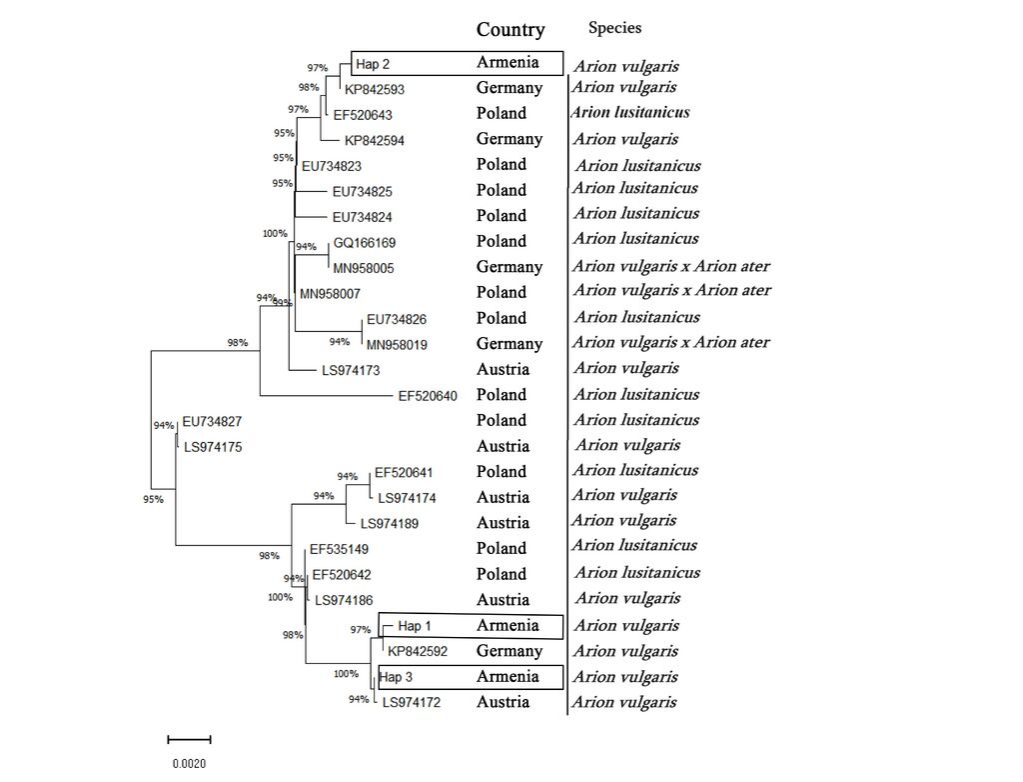
The phylogenetic tree of *A.vulgaris* species, based on the CO1 gene. Hap_1 = Haplotype-1, Hap_2 = Haplotype-2, Hap_3 = Haplotype-3.

Phylogenetic analysis has shown that there are three distant haplotypes of *A.vulgaris* occurring in Gyulagarak Village in Armenia. Haplotype-1 consists of barcoding results of six specimens: CaBOL-1013789, CaBOL-1013790, CaBOL-1013791, CaBOL-1013793, CaBOL-1013795, CaBOL-1013797; Haplotype-2 two specimens: CaBOL-1013792, CaBOL-1013796; and Haplotype-3 only one: CaBOL-1013794. Haplotype-1 and Haplotype-3 are phylogenetically close to each other and to specimens from Germany and Austria. Haplotype-2 is close to specimens from Germany and is phylogenetically further from Haplotype-1 and Haplotype-3 (Fig. 4). This indicates that the species has been introduced from Central Europe. Most of the GenBank records used for the phylogeny in Fig. 4 were for *A.vulgaris* specimens, especially those records from Austria and Germany. For most of the Polish data, taken from Soroka et al. (2009), where the species' anatomical description is similar to *A.vulgaris*, *A.vulgaris* might be misidentified as *A.lusitanicus*. There are three *A.vulgaris* and *A.ater* hybrids – two from Germany and one from Poland – shown in Fig. 4.

## Discussion

Our results confirm the first record of *A.vulgaris* for Armenia. External morphological characters and genital anatomy of the species complied with external and genital traits of *A.vulgaris* (Wiktor 1996, Rowson et al. 2014). However, the bursa copulatrix is usually egg-shaped with a short duct in adults (Wiktor 1996); in our specimens, the bursa was elongated. This difference was probably due to the fact that the collected specimens were not fully mature.

From neighbouring Türkiye, *Arionater* s.l. was first recorded in 2017 (Reise et al. 2018). The taxonomy of some large *Arion* species is extremely problematic due to external morphological similarities and, potentially, hybridisation (Reise et al. 2020). Dissection is required to identify species. The ligula are shorter and can be found in the upper atrium in *Arionater* s.l., but the ligula are elongated and situated in the dilated oviduct in the case of *A.vulgaris*. The specimens from Armenia fit the species description of *A.vulgaris* well, and based on molecular data, Armenian samples are also similar to European *A.vulgaris*. However, the individual photographed near Dilijan, which was not from a garden, but was seen in mixed forest habitat, might suggest *A.ater* occurs in Armenia as well. This is currently unconfirmed though and anatomical and genetic analyses are required before we can say for certain.

*Arionvulgaris* has been confused with *Arionlusitanicus* Mabille, 1868, which is a species endemic to Portugal. After this confusion was disentangled, based on genital morphological differences (Castillejo 1997) and although a molecular study suggests they are separate species (Quinteiro et al. 2005), the name *A.lusitanicus* auct. non Mabille, 1868 was used for a while for the invasive species with a Europe-wide distribution. However, the name *A.vulgaris* had been used more and more frequently for the invasive species, finally becoming its valid name (Balashov 2018, Kadolsky et al. 2018, Reise et al. 2020).

The first specimen of *A.vulgaris* from Armenia was detected in Gyulagarak Village. It was found in mixed gardens where different types of vegetables were planted. Based on our observations, individuals are inactive during the day, but during the night, they actively emerge from their hiding places and start to disperse and feed. An individual representing a possible second population of the species was photographed in 2023 in Dilijan by Prof. S. Pipoyan, but this is an unconfirmed sighting of *A.vulgaris*. The current record of the species from Gyulagarak is the second from the Caucasus (Schikov and Komarov 2021) and the first Arionidae for Armenia.

**Figure 5.**
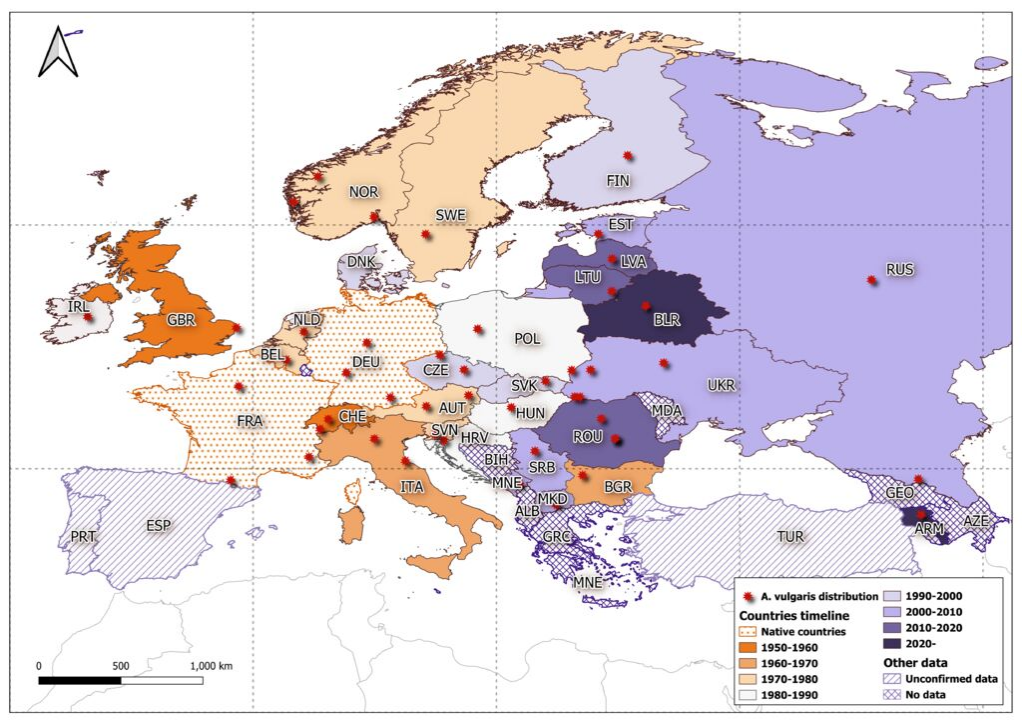
Distribution and dispersal of *A.vulgaris* across Eurasia by decade.

*Arionvulgaris* is a dangerous invasive species that can cause damage to domestic gardens (Proschwitz 1997, Hulme 2009a, Zemanova et al. 2017) and is one of the fastest spreading terrestrial molluscs. In Table 1 and Fig. 5, the distribution of *A.vulgaris* in Eurasia is presented across several decades. However, it should be noted that, for France and Germany, the species is considered to be native (Zemanova et al. 2016).

Based on the map (Fig. 5), it is clear that the species has been spreading in all directions from its native range. It is likely only a matter of time before it will be recorded in Georgia, Azerbaijan, Iran and the eastern part of Türkiye. Considering that species introduction mechanisms have not been recorded, there are several possibilities as to how it could have entered Armenia. If we assume the natural range expansion of the species, it may have entered Armenia from Georgia in the north or from the eastern region of Türkiye to the west. Invasive species are usually understudied, but due to the lack of confirmed records from Georgia and eastern Türkiye, this hypothesis remains unconfirmed. Obtaining haplotype samples from a wider range of locations and a more comprehensive phylogenetic analysis of these could help to determine where the possible routes into Armenia are.

However, a more likley scenario than natural distribution is the human-assisted spread of the species (Meyerson and Mooney 2007). Due to increased international transportation, agricultural or horticultural products provide a fast and convenient way for invasive species to spread (Cowie et al. 2008, Hulme 2009b). Slugs can easily hide in plant pots and a sufficient amount of moisture makes them capable of surviving long-distance travel. This is not just the case for adults; juveniles and unhatched eggs in the soil of plant pots enhance the distribution of invasive molluscs further. As a consequence, botanical gardens and garden centres are potential hotspots for new introductions (Turóci et al. 2023).

Monitoring new invasions of *A.vulgaris* is crucial, as species invasions into new territories can have several negative consequences: for example, the impact on agriculture via reduced yields (Grimm et al. 2000) or on ecosystems as a result of habitat modification and loss of native species (Proschwitz 1997, Knop and Reusser 2012, Zemanova et al. 2017, Reise et al. 2020, Hutchinson et al. 2021). *Arionvulgaris* is also an intermediate host for several cardiopulmonary metastrongyloid nematodes that cause respiratory or systemic disease in canids and felids (Ferdushy et al. 2010, Gismervik et al. 2015, Lange et al. 2018, Penagos-Tabares et al. 2019, Antzée-Hyllseth et al. 2020). Additionally, *A.vulgaris* may also be a potential vector of plant pathogens, such as genus *Phytophthora* de Bary, 1876 (Telfer et al. 2015) and pathogenic bacteria of humans, such as Enterobacteriaceae (Stalder et al. 2014, Gismervik et al. 2015).

However, monitoring inhabited areas and areas into which *A.vulgaris* has possibly been newly introduced can help as a means to survey the population and this enables us to assess whether occupied ecosystems are suitable for this species. We emphasise that prevention of new introductions and eradication of populations of invasive species are very important and provide great challenges for the future.

## Supplementary Material

XML Treatment for Arion vulgaris

## Figures and Tables

**Table 1.** Distribution and first records of *Arionvulgaris* by year around the world. *Note that the Spanish record probably belongs to *A.lusitanicus*, a Portuguese endemic species and not to the invader *A.vulgaris*.

**Country**	**Year of first observation**	**Reference**
France	Native	Moquin-Tandon (1855)
Germany	Native	Schmid (1970)
Spain*	Unconfirmed data	Mabille (1868)
Portugal*	Unconfirmed data	Mabille (1868)
Türkiye	Unconfirmed data	Yildırım and Gürlek (2017)
UK	1954	Kerney (1999)
Switzerland	1956	Turner et al. (1998)
Italy	1965	van Regteren Altena (1971)
Bulgaria	1966	van Regteren Altena (1971)
Slovenia	1970	Wiktor (1996)
Austria	1971	Reischütz and Stojapal (1972)
Norway	1972	Reischütz and Stojapal (1972)
Belgium	1973	Risch and Backeljau (1989)
Netherlands	1973	de Winter (1989)
Sweden	1975	Proschwitz (1989)
Croatia	1983	Wiktor (1996)
Ireland	1984	Anderson (2010)
Hungary	1985	Varga (1986)
Poland	1987	Kozłowski and Kornobis (1994)
Finland	1990	Valovirta (2001)
Czech Republic	1991	Dvořák and Horsák (2003)
Denmark	1991	Proschwitz and Winge (1994)
Slovakia	1992	Reischütz (1994)
Faroe Islands	1996	Bloch (2003)
Montenegro	2002	Vuksa et al. (2003)
Serbia	2002	Vuksa et al. (2003)
Iceland	2003	Ingimarsdóttir and Ólafsson (2005)
Macedonia	2003	Stankovic et al. (2006)
Ukraine	2007	Sverlova and Gural (2008)
Estonia	2009	Palginõmm (2009)
Canada	2009	L’Heureux et al. (2023)
Mexico	2009	Araiza-Gómez et al. (2021)
Russia	2009	Schikov (2016)
Latvia	2010	Rudzīte et al. (2010)
Romania	2012	Papureanu et al. (2014)
Lithuania	2013	Skujienė (2013)
Russia, North Ossetia-Alania	2019	Schikov and Komarov (2021)
Belarus	2021	Ostrovsky (2022)
Armenia	2022	Current Study

## References

[B11119592] Anderson R. (2010). The invasive pest slug *Arionvulgaris* Moquin-Tandon (=*A.lusitanicus* Mabille) (Mollusca: Arionidae) confirmed for Ireland, with an appeal for records. The Irish Naturalists' Journal.

[B11119601] Antzée-Hyllseth H, Trandem N, Torp T, Haukeland S (2020). Prevalence and parasite load of nematodes and trematodes in an invasive slug and its susceptibility to a slug parasitic nematode compared to native gastropods. Journal of Invertebrate Pathology.

[B11119610] Araiza-Gómez V, Naranjo-García E, Zúñiga G (2021). Occurrence in Mexico of two European invasive slug species: *Arionvulgaris* Moquin-Tandon, 1855 and *Arionintermedius* (Norman, 1852. BioInvasions Records.

[B11295667] Balashov I. (2018). Case 3685 – *Arionvulgaris* MoquinTandon, 1855 (Gastropoda, Stylommatophora, Arionidae): proposed validation of the specific name as available. Bulletin of Zoological Nomenclature.

[B11119639] Bloch D. (2003). Morsnigilin ella hin spanski snigilin. Frøði.

[B11119648] Castillejo J. (1997). Las babosas de la familia Arionidae Gray, 1840 en la Península Ibérica e Islas Baleares. Morfología y distribución. Gastropoda, Pulmonata, terrestria nuda). Revista Real Academia Galega de Ciencias.

[B11119657] Cowie R., Hayes K., Tran C., Meyer W (2008). The horticultural industry as a vector of alien snails and slugs: widespread invasions in Hawaii. International Journal of Pest Management.

[B11119666] Davies M. J. (1987). *Arionflagellus* Collinge and *A.lusitanicus* Mabille in the British Isles: A morphological, biological and taxonomic investigation. Journal of Conchology.

[B11120249] de Winter Anton (1989). *Arionlusitanicus* Mabille in Nederland (Gastropoda, Pulmonata, Arionidae). Basteria.

[B11119675] Dvořák L, Horsák M (2003). Současné poznatky o plzáku *Arionlusitanicus* (Mollusca: Pulmonata) v České republice. Časopis Slezského Muzea Opava A.

[B11119684] Ferdushy T., Kapel C. M.O., Webster P., Al-Sabi M. N.S., Grønvold J. R (2010). The effect of temperature and host age on the infectivity and development of Angiostrongylusvasorum in the slug *Arionlusitanicus*. Parasitology Research.

[B11248409] Folmer Ole, Black Mb, Hoeh Wr, Lutz R, Vrijenhoek Robert (1994). DNA primers for amplification of mitochondrial Cytochrome C oxidase subunit I from diverse metazoan invertebrates. Molecular marine biology and biotechnology.

[B11119694] Gismervik K., Aspholm M., Rørvik L. M, Bruheim T., Andersen A., Skaar I. (2015). Invading slugs (*Arionvulgaris*) can be vectors for *Listeriamonocytogenes*. Journal of Applied Microbiology.

[B11119705] Grimm B., Paill W., Kaiser H. (2000). Daily activities of the pest slug *Arionlusitanicus*. Journal of Molluscan Studies.

[B11119714] Hatteland B. A. (2010). Predation by carabid beetles (Coleoptera, Carabidae) on the invasive Iberian slug *Arionlusitanicus*.

[B11119722] Hatteland B. A., Grutle K., Mong C. E., Skartveit J., Symondson W. O.C., Solhøy T (2010). Predation by beetles (Carabidae, Staphylinidae) on eggs and juveniles of the Iberian slug *Arionlusitanicus* in the laboratory. Bulletin of Entomological Research.

[B11119733] Hatteland B. A., Symondson W. O.C., King R. A., Skage M., Schander C., Solhøy T (2011). Molecular analysis of predation by carabid beetles (Carabidae) on the invasive Iberian slug *Arionlusitanicus*. Bulletin of Entomological Research.

[B11119744] Hatteland B. A., Roth S., Andersen A., Kaasa K., Støa B., Solhøy T (2013). Distribution and spread of the invasive slug *Arionvulgaris* Moquin-Tandon in Norway. Fauna Norvegica.

[B11119755] Hulme D. E. (2009). Handbook of alien species in Europe.

[B11119763] Hulme D. E. (2009). Trade, transport and trouble: managing invasive species pathways in an era of globalization. Journal of Applied Ecology.

[B11295543] Hutchinson John M. C., Schlitt Bettina, Reise Heike (2021). One town’s invasion by the pest slug *Arionvulgaris* (Gastropoda: Arionidae): microsatellites reveal little introgression from *Arion*
ater and limited gene flow between infraspecific races in both species. Biological Journal of the Linnean Society.

[B11119772] Iglesias J., Castillejo J., Castro R. (2001). Field Test Using the Nematode Phasmarhabditishermaphrodita for Biocontrol of Slugs in Spain. Biocontrol Science and Technology.

[B11119781] Ingimarsdóttir M, Ólafsson E (2005). Spánarsnigilin finnst á Íslandi, bví miður. Náttúrufræðingurin.

[B11295678] Kadolsky Dietrich, Welter-Schultes Francisco, Bank Ruud A. (2018). Comment (Case 3685) – *Arionvulgaris* Moquin-Tandon, 1855 (Gastropoda, Stylommatophora, Arionidae): modified proposal to preserve the specific name in its accustomed sense. The Bulletin of Zoological Nomenclature.

[B11119790] Kerney M. (1999). Atlas of the land and freshwater molluscs of Britain and Ireland.

[B11295570] Knop Eva, Reusser Nik (2012). Jack-of-all-trades: phenotypic plasticity facilitates the invasion of an alien slug species. Proceedings of the Royal Society B.

[B11119798] Kozłowski J, Kornobis S (1994). *Arion* sp. (Gastropoda: Arionidae) – szkodnik zagrazajacy roslinom uprawnym w wojewodztwie rzeszowskim. Mat. 34 Sesji Nauk. Inst. Ochr. Roœlin, Poznañ.

[B11119807] Kozłowski J (2007). The distribution, biology, population dynamics and harmfulness of *Arionlusitanicus* Mabille, 1868 (Gastropoda: Pulmonata: Arionidae) in Poland. Journal of Plant Protection Research.

[B11119816] Kozłowski J, Jaskulska M, Kozłowska M (2014). Evaluation of the effectiveness of Iron phosphate and the parasitic nematode *Phasmarhabditishermaphrodita* in reducing plant damage caused by the slug *Arionvulgaris* Moquin-Tandon, 1885. Folia Malacologica.

[B11119825] Lange M. K., Penagos-Tabares F., Hirzmann J., Failing K., Schaper R., Van Bourgonie Y. R., Backeljau T., Hermosilla C., Taubert A. (2018). Prevalence of *Angiostrongylusvasorum*, *Aelurostrongylusabstrusus* and *Crenosomavulpis* larvae in native slug populations in Germany. Veterinary Parasitology.

[B11119839] L’Heureux É, Lafond J, Angers B (2023). First record of the invasive slug *Arionvulgaris* Moquin-Tandon, 1885 (Gastropoda, Stylommatophora, Arionidae) in Quebec (Canada). Bioinvasions Records.

[B11119848] Mabille M. J. (1868). Des Limaciens Européens. Revue et magasin de zoologie.

[B11119857] Meyerson Laura A., Mooney Harold A. (2007). Invasive alien species in an era of globalization. Frontiers in Ecology and the Environment.

[B11119866] Moquin-Tandon Alfred (1855). Histoire naturelle des mollusques terrestres et fluviatiles de France. Paris.

[B11119875] Nei M., Kumar S. (2000). Molecular Evolution and Phylogenetics.

[B11119883] Ostrovsky A. M. (2022). New records of synanthropic slugs *Limacusmaculatus* and *Arionvulgaris* (Mollusca, Gastropoda, Stylommatophora) in Belarus. Ruthenica, Russian Malacological Journal.

[B11119892] Palginõmm M (2009). Lusitaanlane luubi all". Eesti Loodus.

[B11119901] Papureanu A. M., Reise H., Varga A. (2014). First records of the invasive slug *Arionlusitanicus* auct. non Mabille (Gastropoda: Pulmonata: Arionidae) in Romania. Malacologica Bohemoslovaca.

[B11119910] Penagos-Tabares Felipe, Groß Katharina M., Hirzmann Jörg, Hoos Christine, Lange Malin K., Taubert Anja, Hermosilla Carlos (2019). Occurrence of canine and feline lungworms in *Arionvulgaris* in a park of Vienna: First report of autochthonous *Angiostrongylusvasorum*, *Aelurostrongylusabstrusus* and *Troglostrongylusbrevior* in Austria. Parasitology Research.

[B11119922] Pfenninger Markus, Weigand Alexander, Bálint Miklós, Klussmann‐Kolb Annette (2014). Misperceived invasion: the Lusitanian slug (*Arionlusitanicus* auct. non‐Mabille or *Arionvulgaris* Moquin‐Tandon 1855) is native to Central Europe. Evolutionary Applications.

[B11120196] Proschwitz T von (1989). *Arionlusitanicus* Mabille - en för Sverige ny snigelart.

[B11120204] Proschwitz T von, Winge K (1994). Iberia skogsnegl - en art pä spredning i Norge (*Arionlusitanicus* Mabille - en anthropochorous slug spreading in Norway). Fauna.

[B11120213] Proschwitz T von (1997). *Arionlusitanicus* Mabille and *A.rufus* (L.) in Sweden. A comparison of occurrence, spread and naturalization of two alien slug species. Heldia.

[B11119931] Quinteiro J., Rodriguez-Castro J., Castillejo J., Iglesias-Piñeiro J, Rey-Méndez M (2005). Phylogeny of slug species of the genus *Arion*: evidence of monophyly of Iberian endemics and of the existence of relict species in Pyrenean refuges. Journal of Zoological Systems.

[B11119941] Rabitsch W (2009). Handbook of Alien Species in Europe.

[B11119949] Rae R., Verdun C., Grewal P. S., Robertson J. F., Wilson M. J. (2007). Biological control of terrestrial molluscs using *Phasmarhabditishermaphrodita* - progress and prospects. Pest Management Science.

[B11119959] Reischütz P, Stojapal F (1972). Bemerkenswerte Mollusken aus Ostösterreich. Mitt. Zool. Ges Braunau.

[B11119968] Reischütz PL, Reischütz PL (1994). Nachrichtenblatt der Ersten Vorarlberger Malakologischen Gesellschaft.

[B11119981] Reise H., Arslangündoğdu Z, Schlitt B, Hutchinson John Michael Christopher, Hızal E, Bacak E. (2018). First records of the terrestrial slug *Arionater* s. l. (Linnaeus, 1758) (Pulmonata: Arionidae) from Turkey. Folia Malacologica.

[B11295552] Reise Heike, Schwarzer Anne-Katrin, Hutchinson John M. C., Schlitt Bettina (2020). Genital morphology differentiates three subspecies of the terrestrial slug *Arionater* (Linnæus, 1758) s.l. and reveals a continuum of intermediates with the invasive *A.vulgaris* Moquin-Tandon, 1855". Folia Malacologica.

[B11120001] Risch P., Backeljau T. (1989). On the occurrence of *Arionlusitanicus* Mabille, 1868 in Belgium (Mollusca: Pulmonata. Annales de la Société Royale Zoologique de Belgique.

[B11120010] Rowson B., Turner J., Anderson R., Symondson B., Rowson B. (2014). Slugs of Britain and Ireland.

[B11120023] Rozas J., Ferrer-Mata A., Sanchez-DelBarrio J., Guirao-Rico S., Librado P., Ramos-Onsins S. (2017). DnaSP 6: DNA Sequence Polymorphism Analysis of Large Data Sets. Molecular Biology and Evolution.

[B11120035] Rudzīte M, Dreijers E, Ozolina-Moll L, Parele E, Rudzītis M, Stalažs A (2010). Latvijas gliemji: Sugu noteicējs. - A Guide to the Molluscs of Latvia. LU Akadēmiskais apgāds.

[B11120046] Schikov Evgeniy V., Komarov Yuriy E. (2021). Detection of an invasive species *Arionvulgaris* Moquin-Tandon, 1855 (Mollusca: Gastropoda: Arionidae) in the Republic of North Ossetia-Alania. Folia Malacologica.

[B11120055] Schikov E. V. (2016). Adventivnye vidy nazemnoy malakofauny tsentra Russkoy ravniny. Ruthenica.

[B11120064] Schmid G. (1970). *Arionlusitanicus* in Deutschland. Archiv für Molluskenkunde.

[B11120073] Skujienė G, Skujienė G (2013). Invasive slugs in Lithuania: results, problems and perspectives of the investigations. Abstracts of the meeting on Slugs and Snails as Invasive Species, IOB/WPRS Slugs and Snails Subgroup.

[B11295606] Soroka M., Kozłowski J., Wiktor A., Kałuski T. (2009). Distribution and genetic diversity of the terrestrial slugs *Arionlusitanicus* Mabille, 1868 and *Arionrufus* (Linnaeus, 1758) in Poland based on mitochondrial DNA. Folia Biol (Krakow).

[B11120087] Stalder Gabrielle L., Loncaric Igor, Walzer Chris (2014). Diversity of enterobacteria including β-lactamase producing isolates associated with the Spanish slug (*Arionvulgaris*). Science of The Total Environment.

[B11120096] Stankovic SV, Stojkoska E, Norris A, Stankovic SV (2006). Annotated checklist of the terrestrial gastropods (Gastropoda) of the Republic of Macedonia. Anniversary Proceedings (1926–2006). Eighty years of achievement by the Macedonian Museum of Natural History.

[B11120110] Sverlova NV, Gural RI, Sverlova NV, Gural RI (2008). First finding of the terrestrial slug *Arion lusitanicus*. – In: Living objects under anthropogenic influence. Abstracts of the 10th International Scientific-Practical Conference.

[B11120124] Tamura K, Nei M, Kumar S, Tamura K (2004). Prospects for inferring very large phylogenies by using the neighbor-joining method. Proceedings of the National Academy of Sciences.

[B11120138] Telfer K. H., Brurberg M. B., Haukeland S., Stensvand A., Talgø V. (2015). Phytophthora survives the digestive system of the invasive slug *Arionvulgaris*. European Journal of Plant Pathology.

[B11120148] Turner H., Kuiper J. G.J., Thew N., Bernasconi R., Rüetschi J, Wüthrich M, Gosteli M. (1998). Fauna Helvetica 2.

[B11120159] Turóci Ágnes, Hutchinson John Michael Christopher, Schlitt Bettina, Reise Heike, Rapala Miklós, Páll-Gergely Barna (2023). Five new introduced terrestrial slugs in Hungary. BioInvasions Records.

[B11120170] Valovirta I Tehokkaan leviämisen mestari Wayback Machine. https://web.archive.org/web/20070609224057/http://www.fmnh.helsinki.fi/elainmuseo/selkarangattomat/tietoa/espanjansiruetana/leviaminen.htm.

[B11120178] van Regteren Altena CO (1971). Neue Fundorte von *Arionlusitanicus* Mabille. Archiv für Molluskenkunde.

[B11120187] Varga A. (1986). Az Arion (Arion) lusitanicus Mabille, 1868 előfordulása Magyarországon (Mollusca) [Arion (Arion) lusitanicus Mabille, 1868 (Mollusca) in Hungary. Folia Historico-naturalia Musei Matraensis.

[B11120222] Vuksa M., Djedovic S., Stojnic B. (2003). IPM approach to control of the slug *Arionlusitanicus* Mabille - a new pest species in Serbia and Montenegro. Slugs & snails: agricultural, veterinary & environmental perspectives. YildirimBCPC Symposium Proceedings.

[B11120231] Wiktor A. (1983). Slugs of Bulgaria (Arionidae, Milacidae, Limacidae, Agriolimacidae - Gastropoda, Stylommatophora). Annales Zoologici.

[B11120240] Wiktor A. (1996). The slugs of the former Yugoslavia (Gastropoda terrestria nuda - Arionidae, Milacidae, Limacidae, Agriolimacidae). Annales Zoologici.

[B11120258] Yildırım MZ, Gürlek ME (2017). Türkiye Karasal Gastropod Faunası için Yeni Kayıt: *Arionvulgaris* Moquin-Tandon 1855 (Gastropoda: Pulmonata: Arionidae. Mehmet Akif Ersoy Üniversitesi Fen Bilimleri Enstitüsü Dergisi.

[B11120267] Zając Kamila S., Hatteland Bjørn A., Feldmeyer Barbara, Pfenninger Markus, Filipiak Anna, Noble Leslie R., Lachowska-Cierlik Dorota (2019). A comprehensive phylogeographic study of *Arionvulgaris* Moquin-Tandon, 1855 (Gastropoda: Pulmonata: Arionidae) in Europe. Organisms Diversity & Evolution.

[B11120288] Zemanova M., Knop E., Heckel G. (2016). Phylogeographic past and invasive presence of *Arion* pest slugs in Europe. Molecular Ecology.

[B11120279] Zemanova Miriam A., Knop Eva, Heckel Gerald (2017). Introgressive replacement of natives by invading *Arion* pest slugs. Scientific Reports.

